# Individual Differences in Human Path Integration Abilities Correlate with Gray Matter Volume in Retrosplenial Cortex, Hippocampus, and Medial Prefrontal Cortex

**DOI:** 10.1523/ENEURO.0346-16.2017

**Published:** 2017-04-17

**Authors:** Elizabeth R. Chrastil, Katherine R. Sherrill, Irem Aselcioglu, Michael E. Hasselmo, Chantal E. Stern

**Affiliations:** 1 Department of Psychological and Brain Sciences and Center for Memory and Brain, Boston University, Boston, MA; 2 Athinoula A. Martinos Center for Biomedical Imaging, Massachusetts General Hospital, Charlestown, MA

**Keywords:** Cerebellum, distance, navigation, rotation, VBM

## Abstract

Humans differ in their individual navigational abilities. These individual differences may exist in part because successful navigation relies on several disparate abilities, which rely on different brain structures. One such navigational capability is path integration, the updating of position and orientation, in which navigators track distances, directions, and locations in space during movement. Although structural differences related to landmark-based navigation have been examined, gray matter volume related to path integration ability has not yet been tested. Here, we examined individual differences in two path integration paradigms: (1) a location tracking task and (2) a task tracking translational and rotational self-motion. Using voxel-based morphometry, we related differences in performance in these path integration tasks to variation in brain morphology in 26 healthy young adults. Performance in the location tracking task positively correlated with individual differences in gray matter volume in three areas critical for path integration: the hippocampus, the retrosplenial cortex, and the medial prefrontal cortex. These regions are consistent with the path integration system known from computational and animal models and provide novel evidence that morphological variability in retrosplenial and medial prefrontal cortices underlies individual differences in human path integration ability. The results for tracking rotational self-motion—but not translation or location—demonstrated that cerebellum gray matter volume correlated with individual performance. Our findings also suggest that these three aspects of path integration are largely independent. Together, the results of this study provide a link between individual abilities and the functional correlates, computational models, and animal models of path integration.

## Significance Statement

Humans vary considerably in their navigational abilities. Differences in brain structure between good and poor navigators could provide critical insight into the brain systems used for successful navigation in humans. This study examined the structural differences that underlie path integration—the updating of position and orientation during movement—which have not yet been tested. This study provides novel evidence that individual differences in gray matter volume in the hippocampus, retrosplenial cortex, and medial prefrontal cortex are related to path integration ability; these regions match the path integration system known from animals. The results link computational and animal models of path integration to human individual differences, providing greater understanding of the navigational system in humans.

## Introduction

Humans differ considerably in their individual navigational abilities, in part because successful navigation relies on several different skills (Wolbers and Hegarty, 2010; Chrastil, 2013). One such capability is path integration, the constant updating of the navigator’s position and orientation during movement (Byrne et al., 2007), particularly in sparse environments without landmarks. Significant individual variability has been observed in path integration abilities (Loomis et al., 1993; Klatzky et al., 1999) and could be linked to individual gray matter volume differences. The goal of this study was to examine the relationship between structural gray matter volumes and variations in path integration abilities in humans, using voxel-based morphometry (VBM).

A number of structural imaging studies, all using landmark-based navigational tasks, have found a relationship between topographical memory and morphology of the hippocampus, with increasing hippocampal volume correlating with better navigational performance (Maguire et al., 2000, 2006; Bohbot et al., 2007; Iaria et al., 2008; Woollett and Maguire, 2011; Hartley and Harlow, 2012; Brown et al., 2014; Wegman et al., 2014). Further correlations have suggested dissociations between place- and response-based strategies and brain structure in hippocampus and striatum (Iaria et al., 2003; Bohbot et al., 2007; Konishi and Bohbot, 2013; Schinazi et al., 2013). Although these studies all suggest a link between hippocampal volume and navigational performance, structural imaging investigations have not examined path integration abilities, which have been shown to rely not only on the hippocampus, but additionally on cortical areas including the retrosplenial cortex (RSC) and medial prefrontal cortex (mPFC; Chrastil et al., 2015, 2016).

Previous research in both animals and humans suggest that hippocampus, entorhinal cortex, RSC, and mPFC are likely candidates to support path integration abilities. Functional imaging studies have demonstrated that hippocampal activity predicts accuracy in navigation in sparse environments (Wolbers et al., 2007; Sherrill et al., 2013). Lesions of the hippocampus and entorhinal cortex have been shown to cause impairments of path integration in rodents (Whishaw et al., 1997; McNaughton et al., 2006; Brun et al., 2008). Activity in both hippocampus and RSC increases with Euclidean distance from the home location (Chrastil et al., 2015), suggesting that these regions support a homing vector mechanism that tracks location during path integration. RSC and hippocampus also track translation and rotation information during virtual self-motion, which are key components of path integration (Chrastil et al., 2016).

RSC activity has been related to tracking heading direction (Baumann and Mattingley, 2010; Marchette et al., 2014), and head direction cells have been found in RSC in rodents (Chen et al., 1994; Cho and Sharp, 2001). Lesions to RSC and nearby posterior parietal cortex in humans and rats cause impairments in recalling directional information (Takahashi et al., 1997; Aguirre and D’Esposito, 1999) and in path integration (Save et al., 2001; Save and Poucet, 2009), suggesting that RSC could be important for orienting in sparse environments. mPFC activity has also been observed during path integration, both when tracking locations and when encoding traveled translations and rotations (Spiers and Maguire, 2007; Wolbers et al., 2007; Sherrill et al., 2013; Arnold et al., 2014; Chrastil et al., 2015). The mPFC’s known contribution to memory and executive function could interact with hippocampal memory systems (Preston and Eichenbaum, 2013; Aggleton, 2014; Ito et al., 2015). In particular, mPFC could contribute to the encoding and maintenance of spatial information—a key element of tracking one’s position through the environment—and therefore could be an important factor during path integration.

We used two behavioral paradigms that probed path integration performance. We developed the tasks for use with functional magnetic resonance imaging (fMRI): one was a location tracking paradigm (Chrastil et al., 2015) and one a paradigm that separately examined both the translational and rotational components of path integration (Chrastil et al., 2016). We then used VBM to relate behavioral differences in those tasks to variation in brain morphology in healthy young adults. One key question this study aimed to address is whether spatial abilities for tracking location, translation, and rotation are all supported by the same brain regions, or whether some aspects of path integration are independent. Based on theoretical and animal models of path integration that focus on the hippocampus, entorhinal cortex, RSC, and mPFC, we predicted that human path integration ability would correlate with greater gray matter volume in these areas.

## Materials and Methods

### Participants

Thirty-one participants were recruited for this study from the Boston University community. Twenty-six participants were included in the final data analysis (mean age 23.69 ± 4.66 SD; 12 males, 14 females). Three participants were not scanned owing to claustrophobia, one participant was found to be ineligible after screening, and one participant fell asleep during the scan as determined by nonresponses for a significant portion of the scanning session. All participants had no history of neurologic disorders. Written informed consent was obtained from each participant before enrollment in accordance with the experimental protocol approved by both the Partners Human Research Committee and the Boston University Charles River Campus Institutional Review Board.

#### Stimuli and tasks overview

**Figure 1.**
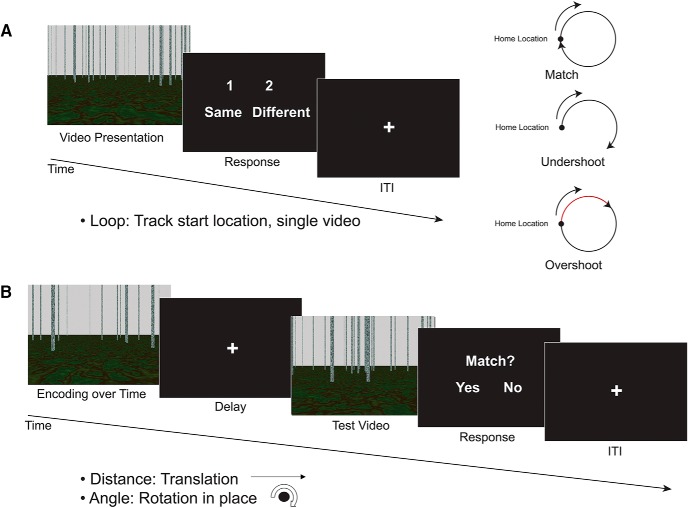
Experimental design (from Chrastil et al., 2015). ***A***, Loop paradigm. A single video was shown with movement along a circle. Participants indicated whether the movement ended in the same location in which it started (match) or if it ended in a different location (nonmatch; undershoots and overshoots were both considered nonmatches). ***B***, Translation and rotation paradigms. Two different videos were presented. First, participants viewed a short encoding video of movement, followed by a delay, then a test video of the same type of movement. Participants indicated whether the movement in the two videos was a match or nonmatch: for example, whether the distance traveled in the two videos was the same. Three experimental tasks were presented in blocks of six trials: loop, distance, and angle.

Complex path integration involves tracking location, often the start or home location. However, complex path integration could be an aggregate of separate translation and rotation computations, and most computational models of path integration require some updating of translation and rotation (Müller and Wehner, 1988; Benhamou et al., 1990; Fujita et al., 1990, 1993). To reflect these different aspects of path integration, two experimental paradigms were presented to participants: a complex location tracking task and simple translation and rotation tasks. Both paradigms required participants to track self-motion during videos shown from a first-person perspective. Detailed information about the stimuli and tasks can be found in our recent fMRI publications introducing these paradigms (Chrastil et al., 2015, 2016). Briefly, in the complex path integration task (loop closure task), participants viewed a single video of movement in a circle in a sparse environment (Fig. 1) and then indicated whether the video ended in the same location in which it started (Chrastil et al., 2015). The translation and rotation tracking tasks (distance and angle tasks) used a modified delayed match to sample (DMS) paradigm to gauge ability (Chrastil et al., 2016). In these two DMS tasks, participants viewed a video of virtual movement, followed by a delay, and then another video presentation. After the second video presentation, participants indicated via a button press whether the movement in the two videos was the same or different. In addition to the experimental tasks, two separate tasks—curve and static image change—were also collected, but are not discussed here. The name of the condition (“Distance,” “Angle,” or “Loop”) was printed in text at the top of the screen during the two video presentations, to ensure that participants were aware of the condition.

The virtual environment was developed using POV-Ray v.3.6, a 3D ray-tracing modeling program. The environment consisted of a textured ground plane with ∼150 textured poles, or “trees,” randomly placed in the scene (Fig. 1). The textured ground and trees in the environment provided optic flow information during the video presentation of movement. The trees were taller than the top of the screen so that height changes could not be used as a cue to distance. The large number of trees and random placement discouraged participants from using the scene arrangement as a landmark, and each video had a different random arrangement of the trees. Movement in the videos never passed directly through a tree. We emphasize that self-motion information used in this study stems purely from visual motion, with no vestibular or proprioceptive input, owing to the constraints of fMRI scanning. Videos of movement in the environment were presented as a series of images, presented at 30 frames/s. The scenes were presented to participants using E-Prime 2.0 (Psychology Software Tools), which also recorded the exact timing of stimulus presentation and participants’ responses.

#### Loop closure task

In the loop closure task, the camera movement in the video traveled in a circular pattern. Once the video ended, participants had to indicate whether the movement in the video ended at the same location in which it started, at the home location (see Chrastil et al., 2015, for more details).

Half of the videos ended in the home location (“match,” a full 360° traversal of the loop) and half were nonmatches, ending at another point along the circle. Half of the nonmatches were undershoots, such that the movement only traversed partway around the circle (225° of the loop). The other half were overshoots, such that movement went past the home location and went partway around a second loop (495° of the loop). Participants were given clear instructions that overshoots were considered nonmatches, and that it was important to determine if the end point itself was the same as the start location. Three different radii of curvature (2.0, 3.0, and 4.5 virtual units) and two different travel speeds (1.5 and 2.0 virtual units/s) were used in the loop task, crossed to yield six angular speeds (0.33, 0.44, 0.50, 0.67, 0.75, and 1.00 radians/s). Length of the videos for the loop task were ∼4–25 s, with an average of 11.5 s. After the video, a response screen was presented, and participants had up to 2 s to respond whether the loop returned to the home location. A 6-s intertrial interval (ITI) began as soon as the response was recorded; thus the duration of the response was based on participants’ reaction time. Loops turned both to the right and to the left in equal numbers; we combined left and right turning direction for analysis.

#### Distance

For both the distance and angle DMS tasks, each trial began with the first “encoding” video, which varied in duration (Chrastil et al., 2016). After the encoding video, a 4-s fixation delay was presented. Next, the second test video was presented, which also varied in duration. Durations of the cue and test videos were varied, based on displays of the speed and the magnitude of the translations and rotations in the virtual environment. Movement was presented at 2 speeds for each of the tasks (1.5 and 2.0 virtual units/s for the distance task and 35° and 40°/s for the angle task). The movement speed in the encoding and test videos matched on half of the trials and did not match on the other half. After the test video, a response screen was presented, and participants had up to 2 s to respond whether the magnitude of the movement was the same or different in the two videos. A 6-s intertrial interval (ITI) began as soon as the response was recorded.

For the distance task, movement in both videos was translation in a straight line. During the response period, participants indicated whether the distance traveled in the test video was the same or different as the encoding video. Two outbound distances were used for the first video, 5 and 9 virtual units. On half of the distance trials, distances in the second video were matches, and half were nonmatches. Half of the nonmatch trials were overshoots of the match distance, and half were undershoots; nonmatches for the 5-unit encoding video were either 2 or 9 units, and nonmatches for the 9-unit encoding video were either 5 or 13 units. The length of the videos varied based on distance and speed presented, with an average of 4 s across all trials.

#### Angle

Movement in both videos was rotation in place, similar to a person standing in a single location and turning in place. During the response period, participants indicated whether the rotation angle in the test video was the same or different as the encoding video. Two degrees of rotation were used for the first video, 80° and 140°. In half of the trials, the rotations in the second video were matches to the cue video, and half of the trials were nonmatches. Half of the nonmatch trials were overshoots of the match rotation, and half were undershoots; nonmatches for the 80° encoding video were either 40° or 120°, and nonmatches for the 140° encoding video were either 80° or 200°. Left and right turns were equally represented across all rotation trials, and the encoding and test video always went in the same direction; left and right rotation trials were collapsed in our analysis. The length of the videos varied based on degree of rotation and speed presented, with an average of 3 s.

### Procedure

#### Prescan training

Participants were trained outside the scanner the day before scanning. Participants were given a general description of movement in the environment and shown a short example. They were then given specific instructions and several practice runs with feedback for each of the tasks in turn.

#### Spatial abilities testing

At the conclusion of the prescan training, participants completed several spatial abilities tasks, which allowed us to examine potential individual differences. These abilities tasks included the self-report questionnaire Santa Barbara Sense of Direction Scale (Hegarty et al., 2002), a questionnaire about frequency and manner of personal video game use, the Road Map Test in which participants report the direction of each turn in a route predrawn on a city map (Money and Alexander, 1966; Zacks et al., 2000), and the Perspective-Taking Spatial Orienting Test in which participants view a 2D array of objects on a page and indicate directional relationships from imagined viewpoints (Kozhevnikov and Hegarty, 2001).

#### Experimental task

While the structural scans were being acquired, participants were given a practice run with feedback using examples from the training, with eight trials per task block. After practice, there were six functional test runs, randomized across participants, for a total of 36 trials per condition. Each of the test runs consisted of one block each of the experimental tasks (distance, angle, curve, loop, and static image change). Each block contained six trials of the task, with match and nonmatch trials counterbalanced across runs. The task order of each block was counterbalanced across runs. Length and direction of movement, as well as speed of travel, were counterbalanced across conditions and runs. Because the ITI began as soon as participants made their responses, the scan time for each of the six runs varied somewhat, but generally lasted just under 10 min. Total scan time for the experimental task was ∼1 h.

### MRI acquisition

Images were acquired at the Athinoula A. Martinos Center for Biomedical Imaging, Massachusetts General Hospital in Charlestown, MA, using a 3-Tesla Siemens Magnetom TrioTim scanner with a 32-channel Tim Matrix head coil. High-resolution T1-weighted multi-planar rapidly acquired gradient echo (MP-RAGE) structural scans were acquired using Generalized Autocalibrating Partially Parallel Acquisitions (GRAPPA; TR, 2530 ms; TE, 3.31 ms; flip angle, 7°; slices, 176; resolution, 1 mm isotropic). Two high-resolution T1-weighted images were acquired and visually inspected for motion or other scanning artifacts, with the higher-quality image used for analysis. If no artifacts were found, the first image acquired was selected for analysis.

### Behavioral analysis

Behavioral performance was assessed using MatLab (MathWorks) and SPSS20 (IBM). Within-subjects repeated-measures ANOVAs were used to assess potential differences in accuracy and reaction time between the different conditions. Pearson correlations were also conducted to assess the relationship between accuracy on each of the experimental tasks and accuracy on the other experimental tasks and measures of spatial ability.

### Voxel-based morphometry

Gray matter volume was analyzed using standard VBM methods in SPM8 (Wellcome Department of Cognitive Neurology, London, UK). Structural images were segmented using SPM8’s New Segment option into gray matter, white matter, and cerebral spinal fluid (CSF) images and were bias-corrected. Gray matter segmentation images were spatially normalized into standard Montreal Neurologic Institute space using the Diffeomorphic Anatomic Registration Through Exponentiated Lie algebra (DARTEL) algorithm (Ashburner, 2007) for a high degree of intersubject registration. Gray matter images were resampled during normalization (1.5 mm^3^ isotropic voxels) and spatially smoothed using a 6-mm full-width at half-maximum Gaussian kernel. VBM analyses were conducted using standard “modulated” smoothed gray matter images, providing a measure of regional gray matter volume (Mechelli et al., 2005).

### VBM statistical analysis

Proportion correct of each of the three experimental tasks for each participant were entered as covariates with smoothed gray matter volume estimate images into a second-level multiple regression analysis in SPM8. *t* statistic images, representing the strength of the linear association, were calculated in SPM. Significant positive relationships indicating local gray matter volume estimates were predicted by accuracy in the task of interest. Individual participant age, sex, and total brain volume were included as additional covariates for the regression analysis to control for their potentially confounding influence on brain structure and performance. Both positive correlations (indicating better performance) and negative correlations (indicating poorer performance) were tested. A correlation ρ of 0.5 with α = 0.05 and 0.8 power yields a sample size of 23 with a critical *r* of 0.35 (see Hartley and Harlow, 2012 for an example of expected effect size). Thus, our sample size of 26 was deemed sufficient for this study.

Region of interest (ROI) and whole-brain analyses were performed for each regression analysis. Based on human and animal literature, we had strong *a priori* hypotheses that the hippocampus contributes to path integration and human short-term memory in a DMS task (Stern et al., 1996; Schon et al., 2004; Wolbers et al., 2007; Newmark et al., 2013; Sherrill et al., 2013; Brown et al., 2014; Howard et al., 2014), that RSC contributes to spatial orientation (Cho and Sharp, 2001; Baumann and Mattingley, 2010; Marchette et al., 2014), that entorhinal cortex contributes to path integration (McNaughton et al., 2006; Brun et al., 2008), that the parahippocampal cortex contributes to human navigation and path integration (Epstein and Vass, 2013; Sherrill et al., 2013), and that the mPFC is involved in tracking location and encoding spatial information (Spiers and Maguire, 2007; Wolbers et al., 2007; Sherrill et al., 2013; Arnold et al., 2014).

To test these hypotheses, we created an ROI mask from the anatomic boundaries of the left and right hemisphere hippocampi, left and right mPFC (superior medial PFC), and the entire left and right parahippocampal gyri (including entorhinal cortex) using the Wake Forest University (WFU) Pick-Atlas automatic anatomic labeling (aal; Tzourio-Mazoyer et al., 2002; Maldjian et al., 2003) available for SPM. The WFU Pick-Atlas does not have an anatomic ROI for the RSC, so we generated an ROI using the anatomic tracing program ITK-SNAP (Yushkevich et al., 2006). This ROI tracing followed along the anatomic boundaries and Brodmann areas outlined in Vann et al. (2009) and Damasio (2005), including the extreme posterior cingulate, the cingulate isthmus connecting to the parahippocampal gyrus, and the most ventral and posterior areas of the precuneus, without extending into the occipital-parietal sulcus. The border between the RSC and PHC was defined as the first slice where the hippocampus tail was visible, since the hippocampus tail serves as the boundary marker for PHC (Pruessner et al., 2002). It is important to note that this was an anatomically defined RSC ROI and included some, but not all, areas of the broader, functionally defined retrosplenial complex (Epstein, 2008). We combined the hippocampal, parahippocampal gyrus, mPFC, and RSC ROIs and resampled to the appropriate image space in SPM. We applied a voxel-wise statistical threshold of *p* < 0.05 to the contrast maps. To correct for multiple comparisons, we applied a cluster-extent threshold technique. The updated 3dFWHMx program in the AFNI software package (version 16.0.01) was used to derive estimates of smoothness of the ROI, yielding an average estimate of 6.437 mm. The 3dClustSim program in AFNI was used to conduct a 10,000-iteration, 6.437-mm autocorrelation Monte Carlo simulation of the ROI volume (29,583 voxels, resampled to 1.5-mm^3^ space); a minimum voxel extent of 336 was determined to maintain a family-wise error rate of *p* < 0.05. Where possible, we also report when clusters within our ROI held at more conservative voxel-wise significance levels, using the following voxel-wise thresholds: *p* < 0.01 (minimum 130 voxels), *p* < 0.005 (minimum 92 voxels), and *p* < 0.001 (minimum 44 voxels), where the cluster minimums indicate a cluster significance of *p* < 0.05. For the ROI analyses, only voxels within the ROI were examined, meaning that the minimum cluster extent had to be met entirely within the ROI. Whole-brain analysis was used to determine clusters that extended beyond the ROI boundaries or elsewhere in the brain.

The whole-brain analysis used the voxel space of the entire brain, and so a separate Monte Carlo simulation was used to determine the cluster threshold. For this analysis, a voxel-wise statistical threshold of *p* < 0.01 was applied to the whole-brain contrast maps. Similar to the ROI analysis, 3dFWHMx was used to derive estimates of smoothness of the whole brain ResMS header file, yielding an average estimate of 7.693 mm. 3dClustSim was used to conduct a 10,000-iteration, 7.693-mm autocorrelation Monte Carlo simulation analysis on voxels within the group functional brain space using the ResMS header file (348,027 total voxels). From this analysis, a minimum voxel extent of 333 was determined to maintain a family-wise error rate of *p* < 0.05 (voxel-wise *p* < 0.01). In the results tables, we also report which areas within the whole brain held at the more conservative voxel-wise significance level of *p* < 0.001 (minimum 120 voxels, cluster corrected to *p* < 0.05). We used Damasio (2005) and Pruessner et al. (2000, 2002) as references for localization in the cortex and Schmahmann et al. (1999) for the cerebellum. For visualization purposes, gray matter volumes were extracted from 5-mm spheres centered on peak coordinates in our ROIs and plotted against proportion correct in the task, with regression lines based on the full model.

## Results

### Behavioral performance

Mean proportion correct for the experimental tasks were as follows: distance, 0.677 (SEM ± 0.020); angle, 0.702 (± 0.029); loop, 0.593 (± 0.022). One-sample *t* tests found that the means for all three tasks were significantly greater than the chance level of 0.5 (distance: *t*_25_ = 8.954, *p* < 0.001; angle: *t*_25_ = 7.017, *p* < 0.001; loop: *t*_25_ = 4.297, *p* < 0.001). Two participants scored below 0.5 in the distance task, as did two in the angle task, and five in the loop task. However, none of the participants had a proportion correct of <0.5 in more than one task. Because of this finding, that low proportion correct was related to individual tasks rather than overall poor performance, and because our VBM statistics included all three behavioral tasks in a single model, all 26 participants were included in the remaining analyses.

A within-subjects repeated-measures ANOVA found that the only statistical differences in performance between the experimental conditions was that the distance and angle tasks were more accurate than the loop task (overall ANOVA main effect of task: *F*_2,50_ = 7.404, *p* = 0.002, η_p_^2^ = 0.228; post hoc tests: distance versus angle, *p* = 1.000; distance versus loop, *p* = 0.007; angle versus loop, *p* = 0.011, Bonferroni corrected). Mean reaction times were as follows: distance, 868.05 ms (± 25.65); angle, 852.18 ms (± 23.61); loop, 893.57 ms (± 22.10). There were no differences between tasks for reaction time (main effect of task: *F*_2,50_ = 2.463, *p* = 0.095, η_p_^2^ = 0.090, all post hoc pairwise *p* > 0.1, Bonferroni corrected).

Surprisingly, performance on the tasks appeared to be largely independent. There were no correlations between accuracy between any of the experimental tasks (distance-angle: *r*_24_ = 0.301, *p* = 0.135; distance-loop: *r*_24_ = 0.287, *p* = 0.156; angle-loop: *r*_24_ = 0.113, *p* = 0.583), suggesting that abilities on the translational and rotational components of path integration and complex location tracking are unrelated to each other. There were marginal relationships between proportion correct in the angle task and scores on the Perspective-Taking Test (*r*_24_ = –0.372, *p* = 0.061, lower Perspective-Taking scores indicate better performance) and the Road Map Test (*r*_24_ = 0.349, *p* = 0.081, higher Road Map score indicate better performance), but no other relationships between the spatial abilities measures and the experimental tasks were seen (all *p* > 0.1). These results suggest that path integration abilities might be independent of landmark-based navigation.

There was no relationship between age and proportion correct on any of the tasks (all *p* > 0.6). In addition, there was no difference in accuracy between males and females on the distance (*r*_24_ = –0.492, *p* = 0.628) or angle (*r*_24_ = 0.234, *p* = 0.817) tasks, although women were marginally more accurate on the loop task then men (*r*_24_ = –2.016, *p* = 0.055; male mean, 0.549; female mean, 0.631).

We also completed a more detailed analysis of different conditions within the behavioral data. For the loop task, participants were significantly more accurate for trials in which the loop did not end at the home location (nonmatch) than for trials that ended at the home location (match; paired *t* test, *t*_25_ = –4.147, *p* < 0.001), but they were not faster in their responses (*t*_25_ = 1.134, *p* = 2.67). Participants were faster in responding when the loops in nonmatch trials were overshoots compared with undershoots (paired *t* test, *t*_25_ = –6.987, *p* < 0.001), suggesting that participants were prepared to respond for the overshoots, although they were not more accurate (*t*_25_ = –1.392, *p* = 0.176). A repeated-measures ANOVA found no differences in accuracy (*F*_2,50_ = 0.539, *p* = 0.587, η_p_^2^ =0.021) or reaction time (*F*_2,50_ = 0.090, *p* = 0.914, η_p_^2^ = 0.004) between the three loop sizes, nor was there a difference in accuracy or reaction time between the two travel speeds (paired *t* tests: accuracy *t*_25_ = –0.303, *p* = 0.764; RT *t*_25_ = 0.466, *p* = 0.645).

In the distance task, paired *t* tests found that participants were both more accurate (*t*_25_ = –2.838, *p* = 0.009) and faster (*t*_25_ = 2.225, *p* = 0.035) in nonmatch trials than in match trials. There was no difference in accuracy between nonmatch overshoots and undershoots (*t*_25_ = 0.940, *p* = 0.356), but participants were faster in the overshoots (*t*_25_ = –5.492, *p* < 0.001), again suggesting that participants were prepared to make a response for the overshoots. Participants were more accurate (*t*_25_ = 2.215, *p* = 0.044) and faster (*t*_25_ = –2.110, *p* = 0.045) in the distance task when the speeds in the two videos matched compared with when they did not match. Finally, participants tended to be more accurate (*t*_25_ = 3.191, *p* = 0.004) when the first video had a shorter distance, but there were no effects on reaction time (*t*_25_ = 0.255, *p* = 0.800).

For the angle task, paired *t* tests found no increase in accuracy for nonmatch trials compared with match trials (*t*_25_ = –0.594, *p* = 0.558), although participants were marginally faster (*t*_25_ = 1.992, *p* = 0.057). Participants were more accurate for undershoots (*t*_25_ = 2.493, *p* = 0.020) but were faster for overshoots (*t*_25_ = 9.198, *p* < 0.001). Participants were also more accurate when the speeds in the two videos matched (*t*_25_ = 2.244, *p* = 0.034), but they were not faster in their responses (*t*_25_ = 0.206, *p* = 0.839). Finally, participants were both faster (*t*_25_ = 3.127, *p* = 0.004) and more accurate (*t*_25_ = –2.229, *p* = 0.035) when the first video had the larger angle.

In summary, this detailed analysis shows a trend for faster reaction time when the trial overshot the target, suggesting that participants were aware of the overshoot and were prepared to respond. Nonmatch trials tended to be easier to detect in general. For the distance and angle tasks, matching speeds in the two videos also facilitated performance, although participants were well above chance even in trials of nonmatching speeds.

### VBM results

For ROI analysis, a *p* < 0.05 voxel-wise significance threshold was maintained, corrected to a family-wise significance level of *p* < 0.05 using a cluster correction threshold of a minimum of 336 contiguous voxels. Whole-brain results had a voxel-wise threshold of *p* < 0.01, cluster corrected to family-wise *p* < 0.05, with a minimum cluster extent of 333 voxels. Here we report the *xyz* coordinate of the peak voxel in each cluster, the uncorrected *t* value and *p* value of the peak voxel, and the cluster size (*k*) in voxels. Table 1 summarizes whole-brain results for all tasks, including the size of the significant cluster, and MNI coordinate and *t* value of the peak voxel.

**Table 1.** Whole-brain results of VBM during the experimental tasks: loop closure, distance tracking, and angle tracking

Contrast	Brain region	Left			Right		
		*k*	*t*	MNI *x*,*y*,*z*	*k*	*t*	MNI *x*,*y*,*z*
Loop							
Correlation with better performance	vmPFC/gyrus rectus	335	6.11*	–2,14,–20	335	3.53	3,14,–18
	Medial prefrontal cortex				1127	4.35	12,54,9
	Cingulate (anterior)	1127	4.29	–8,32,–8		3.85	3,42,–3
	Putamen	617	4.20	–26,12,–11			
	Sylvian fissure	370	5.15	–42,–12,–12			
	Retrosplenial cortex	373	3.77	–6,–54,20			
	Cuneus				373	3.39	8,–72,22
	Lateral occipital gyrus	525	5.19*	–21,–96,–9			
Correlation with worse performance	Cerebellum lobule III	669	4.47	–3,–56,–10	669	3.27	2,–45,–12
	Cerebellum lobule IV		3.58	–6,–47,–6		4.88*	2,–47,–6
	Cerebellum lobule IX				973	5.63*	20,–38,–50
	Cerebellum lobule VIIB					3.71	35,–42,–48
Distance							
Correlation with better performance							
Correlation with worse performance							
Angle							
Correlation with better performance	Cerebellum lobule IX				1782	3.88	8,–62,–36
	Cerebellum lobule VIIIB					4.39	20,–62,–45
	Cerebellum crus I	737	4.01	–41,–42,–39		3.77	18,–83,–20
	Cerebellum crus II					3.32	12,–70,–38
Correlation with worse performance	vmPFC/gyrus rectus				536	3.53	5,27,–23
	Orbitofrontal cortex					4.29	12,48,–20
	Inferior frontal gyrus				1749	3.77	44,24,–6
	Sylvian fissure	630	5.90	–42,6,4		3.99	42,8,11
	Superior temporal gyrus					3.96	48,4,–4
	Insula (anterior)		4.19	–38,18,2		3.22	32,26,–2
	Intraparietal sulcus	1319	5.59*	–21,–81,29			
	Parietal-occipital sulcus		4.80	–18,–74,38			

Correlations of local gray matter volumes with good and poor performance are listed for all tasks, with the size of the cluster (*k*), and *t* value and MNI coordinate of the peak voxel. These values reflect a voxel-wise statistical threshold of *p* < 0.01 corrected to family-wise *p* < 0.05 with a minimum cluster threshold of 333 voxels.

* Result holds at the voxel-wise *p* < 0.001 level (family-wise *p* < 0.05, minimum cluster threshold of 120 voxels).

#### Loop closure

**Figure 2.**
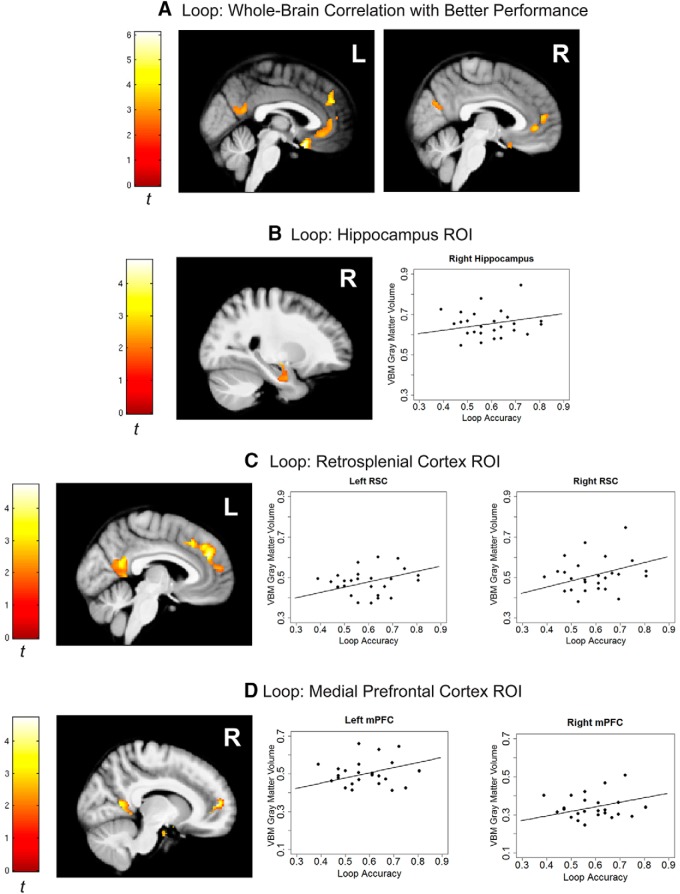
VBM results for the loop task. ***A***, Whole-brain results for positive correlation between gray matter volume and accuracy (proportion correct) in the loop task. Significant correlations were found in retrosplenial cortex, medial prefrontal cortex, anterior cingulate, vmPFC/gyrus rectus, cuneus, putamen (not shown), sylvian fissure (not shown), and lateral occipital gyrus (not shown). Whole-brain results are thresholded at voxel-wise *p* < 0.01, cluster correction of 333 voxels to family-wise *p* < 0.05. ***B***, ROI showing the hippocampus (*r* = 0.165). ***C***, ***D***, ROI showing the RSC (left *r* = 0.261; right *r* = 0.303) and mPFC (left *r* = 0.273; right *r* = 0.239). For visualization purposes, gray matter volumes were extracted from 5-mm spheres centered on peak coordinates in our ROIs and plotted against proportion correct in the task, with regression lines and *r* values based on the full model. ROI thresholded at voxel-wise *p* < 0.05, cluster correction of 336 voxels to family-wise *p* < 0.05.

ROI analysis found a significant positive relationship between accuracy in the loop task and local gray matter volume estimates in the right hippocampus (Fig. 2; *xyz*, 27,–8,–26; *t*_25_ = 2.13; *p* = 0.0232; *k* = 560), which extended into the amygdala (*xyz*, 24,–9,–12; *t*_25_ = 2.58; *p* = 0.0093; *k* = 560). A further positive relationship between performance in the loop task and gray matter volume in both left and right RSC was observed (Fig. 2; *xyz*, –6,–54,20; *t*_25_ = 3.77; *p* = 0.00064; *k* = 811; *xyz*, 14,–51,10; *t*_25_ = 3.57; *p* = 0.001; *k* = 629). In addition, a region spanning bilateral mPFC also showed this correlation between gray matter volume and accuracy in the loop task (*xyz*, –3,44,29; *t*_25_ = 4.72; *p* = 0.000075; *k* = 1541; *xyz*, 12,54,9; *t*_25_ = 4.35; *p* = 0.00017; *k* = 1541). This relationship was upheld at more conservative voxel-wise thresholds bilaterally in both RSC (left: *p* < 0.005, *k* = 105; right: *p* < 0.01, *k* = 183) and mPFC (left: *p* < 0.001, *k* = 58; right: *p* < 0.01, *k* = 156).

At the whole-brain level, the relationship between gray matter volume and accuracy was upheld in left RSC and right mPFC (Fig. 2). Additional positive relationships between accuracy and gray matter volume were observed in bilateral anterior cingulate, bilateral vmPFC/gyrus rectus, left putamen, left Sylvian fissure, right cuneus, and left lateral occipital gyrus (Table 1).

Significant negative relationships between accuracy in the loop task and local gray matter volume estimates were observed only at the whole-brain level in the cerebellum, corresponding medially to bilateral lobule III extending to bilateral lobule IV, and laterally in right lobule VIIB extending to lobule IX (Table 1).

#### Distance

The ROI showed no significant relationships in either the positive or negative direction for the distance task. There were also no positive or negative relationships at the whole-brain level.

#### Angle

**Figure 3.**
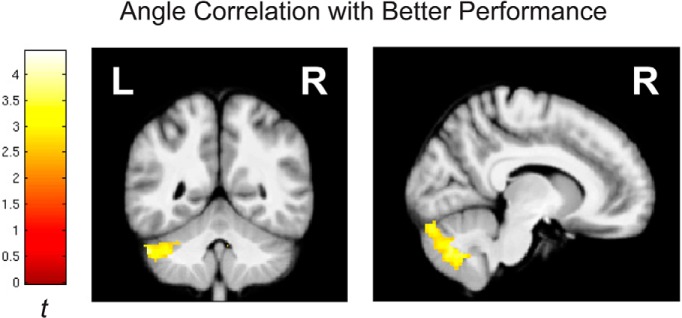
VBM results for the angle task. Positive correlation between gray matter volume and accuracy (proportion correct) at the whole-brain level. Whole-brain results are thresholded at voxel-wise *p* < 0.01, cluster correction of 333 voxels to family-wise *p* < 0.05.

For the angle task, the only significant positive relationship between accuracy and local gray matter volume estimates was observed at the whole-brain level in the cerebellum (Fig. 3). The cerebellar gray matter differences were located bilaterally in crus I, with a cluster including crus II, lobule IX, and lobule VIIIB also showing gray matter relationships on the right (Table 1).

A significant negative relationship was observed in the ROI between both right hippocampus head (*xyz*, 26,–10,–20; *t*_25_ = 2.80; *p* = 0.0057; *k* = 505) and left mPFC (*xyz*, –2,32,38; *t*_25_ = 4.61; *p* = 0.000096; *k* = 562) and performance in the angle task. At more conservative thresholds, the relationship held in left mPFC (*p* < 0.005, *k* = 92), and a relationship with right RSC was observed at *p* < 0.01 (*xyz*, 20,–51,16; *t*_25_ = 3.37, *p* = 0.0016; *k* = 136). At the whole-brain level, negative relationships were also observed in right orbitofrontal cortex, bilateral Sylvian fissure extending into inferior frontal gyrus on the right, and a cluster spanning left intraparietal sulcus and left parietal-occipital sulcus (Table 1).

## Discussion

This experiment examined individual morphologic differences in brain regions that support human path integration. We conducted VBM to relate local gray matter volume to several path integration abilities: tracking a home location and estimating the translational and rotational components of path integration. Consistent with our hypothesis, gray matter volume in the RSC, hippocampus, and mPFC positively correlated with performance in complex path integration tracking a home location. Further, volumetric differences in the cerebellum positively correlated with performance in the angle task. These findings provide novel evidence that individual differences in gray matter volume in hippocampus, RSC, and mPFC contribute to the ability to track location during path integration.

The relationship between gray matter volume and individual abilities has been observed in a number of cognitive domains, including examinations of skilled musicians (Gaser and Schlaug, 2003), experienced taxi drivers (Woollett and Maguire, 2011), exercise training regimens (Erickson et al., 2011), and acquisition of video game skills (Erickson et al., 2010). Findings from these studies and the present experiment suggest that larger brain volumes in specific regions are related to individual abilities; however, the mechanisms behind these relationships have not fully been determined. Innate differences in volume or neural plasticity due to consistent use and training could underlie these effects. The participants in the present study have not to our knowledge had specialized training relating to path integration, although some participants could have more experience with navigation or other factors that could contribute to navigational ability. It is important to note that there was minimal relationship between the navigational abilities measures (SBSOD, Perspective-Taking Test, Road Map Test) and the three path integration tasks, suggesting that path integration skills could be independent of landmark-based navigation. Moreover, accuracy levels on the three path integration tasks were not related to each other. This finding suggests that tracking location, tracking translation, and tracking rotation during self-motion could involve different aspects of path integration and that path integration is composed of multiple task-specific abilities. This study provides strong evidence of the relationship between navigational abilities and gray matter volume that is specific to path integration.

### Gray matter volume in retrosplenial cortex corresponds to successful location tracking

We found a significant positive correlation between gray matter volume in RSC and accuracy in the loop task, indicating that greater neural resources in RSC made a significant contribution to tracking locations during complex path integration. Individuals with greater gray matter volume in RSC might be better able to track their distance from the home location, possibly due to an increased number of neurons or reorganization of the RSC microstructure. Our corresponding functional imaging paradigm complements this finding: RSC activity increased with increasing distance from the home location and had greater BOLD signal for correct path integration trials than for incorrect trials (Chrastil et al., 2015), suggesting that this region plays a role in tracking distance during path integration. Here, we show that individual differences in gray matter volume correspond to ability in the loop closure task, providing a coda to the functional account of RSC contributions to human path integration.

The current results provide the first evidence of a significant relationship between path integration abilities and gray matter volume in RSC. These morphologic findings are supported by functional imaging studies that have found that navigating in sparse environments, which relies on path integration mechanisms, recruits RSC (Sherrill et al., 2013; Chrastil et al., 2015, 2016). Larger gray matter volume in RSC could indicate greater neural resources for recruitment during functional imaging. RSC is also sensitive to heading direction in both humans and animals (Chen et al., 1994; Cho and Sharp 2001; Baumann and Mattingley 2010; Marchette et al., 2014), but is also active during many landmark-based navigation tasks (Hartley et al., 2003; Rosenbaum et al., 2004; Wolbers et al., 2004; Wolbers and Büchel, 2005; Zhang and Ekstrom, 2013), indicating that this region subserves a number of navigational functions. In a landmark-based navigation task, Schinazi et al. (2013) found a correspondence between pointing accuracy and RSC gray matter volume. However, the results presented here suggest that RSC makes a significant contribution to path integration processes such as tracking a location in a sparse environment. Together, these findings suggest that path integration may be important for tracking metric information, even in a landmark-rich environment.

The current findings support the idea that RSC not only selectively processes egocentric heading signals needed for path integration, but also that it does not contribute equally to all aspects of path integration. There was no relationship between RSC volume and the distance or angle tasks. However, functional data using the same paradigm shows that increased RSC activity during maintenance of rotational information in the angle task predicted better performance (Chrastil et al., 2016). Thus, although there was no relationship between volume of RSC and accuracy, good navigators did show functional differences compared with poor navigators, suggesting that RSC plays a key role for location tracking and is also important for the maintenance of orientation information.

### Hippocampal volume and successful path integration

Greater gray matter volume in right anterior hippocampus corresponded to increased accuracy in the loop task. Notably, several studies using landmark-based navigation tasks have observed a positive correlation between gray matter volume in the hippocampus and metric survey knowledge, not just for route-based navigation (Maguire et al., 2000; Woollett and Maguire, 2011; Hartley and Harlow, 2012; Schinazi et al., 2013). Metric information may be encoded through path integration to create survey knowledge (Gallistel, 1990; Chrastil, 2013). Thus, converging findings from the present path integration task and landmark-based tasks that probe metric survey knowledge suggest that hippocampal volume could be a common link between spatial coding in path integration and overall topographical knowledge. This link is further strengthened by a recent study showing that hippocampal and entorhinal cortex gray matter volume is associated with better performance on a goal-directed navigation task in a sparse environment that required updating position and orientation while keeping track of the overall layout (unpublished data).

These results are consistent with areas that demonstrated functional activation in the loop task. We found hippocampal activity to be associated with homing vector distance from the home location, suggesting that the hippocampus tracks homing distance, similar to RSC (Chrastil et al., 2015). Other functional imaging studies have also shown a relationship between hippocampal activity and distance to a goal location (Spiers and Maguire, 2007; Morgan et al., 2011; Viard et al., 2011; Sherrill et al., 2013; Howard et al., 2014). In addition, patients with lesions to the hippocampus and other MTL areas have shown impairments in path integration—in particular distance estimations—highlighting hippocampal contributions to path integration (Worsley et al., 2001; Philbeck et al., 2004; Yamamoto et al., 2014). Our VBM results supplement this finding, suggesting that navigators with larger hippocampal gray matter volume may be better able to track distance from the home location, leading to increased accuracy.

### Prefrontal cortex volume and navigational circuits

mPFC gray matter volume, including the anterior cingulate cortex, was positively correlated with performance in the loop task. mPFC was also functionally recruited during successful navigation of the loop task, but unlike hippocampus and RSC, mPFC did not track location information (Chrastil et al., 2015). This finding suggests that the mPFC contributions to encoding location information for successful path integration differ from those of hippocampus and RSC. In humans, mPFC has been shown to demonstrate greater activity during navigation when the start and goal locations are close to each other (Viard et al., 2011), is recruited by navigators who perform well at path integration (Arnold et al., 2014), and has been observed in other navigation tasks that require learning the location of a goal (Spiers and Maguire, 2007; Sherrill et al., 2013). In rodents, neurons recorded in mPFC code for spatial goals (Hok et al., 2005). Together, these findings indicate that this region is sensitive to goal-directed information and could contribute to spatial working memory.

The mPFC receives strong anatomic connections from anterior hippocampus (Aggleton, 2014), but the reciprocal connections from mPFC to hippocampus go through RSC or the nucleus reunions of the thalamus (Aggleton, 2014; Miller et al., 2014; Ito et al., 2015). The results of this study are consistent with animal models of this circuit (Blankenship et al., 2016) and moreover suggest that individual gray matter volume of hippocampal, RSC, and mPFC regions within this circuit are related to the ability to track a goal location. These results are also consistent with computational models that show how information about direction and speed of movement from head-direction cells in RSC and other regions could update grid cell and hippocampal place cell responses, and subsequently update goal information in PFC (Burgess et al., 2007; Byrne et al., 2007; Hasselmo, 2009; Erdem and Hasselmo, 2012). Our results therefore provide a critical link between the individual differences observed in human navigation and functional correlates, computational models, and animal models of path integration.

### Cerebellar volume corresponds to rotational accuracy

The results from the angle task revealed that accuracy was significantly correlated with gray matter volume in the cerebellum, centering on crus I and extending into crus II, with lobules VIIIB and IX also showing a correspondence. During a spatial navigation task, crus I has been shown to be active and functionally connected to navigationally important regions including the hippocampus, medial parietal cortex, and mPFC (Iglói et al., 2014). Crus I has also been shown to be active during working memory, whereas crus II is active during mental rotation (Stoodley, 2012; Stoodley et al., 2012). Crus I and particularly crus II have direct connections with primate dorsolateral prefrontal cortex (dlPFC; Kelly and Strick, 2003; Ramnani, 2006) and are functionally connected to the fronto-parietal network (Sang et al., 2012) and default mode network (Stoodley, 2012). In support of these connections, our functional study demonstrated increased BOLD activation in the right dlPFC during successful encoding of the angle task, (Chrastil et al., 2016). Together, these results suggest that the cerebellum and dlPFC together process rotational signals.

### Spatial abilities

The results of this study provide several new insights into the relationships between different spatial abilities. We were surprised to find that the three experimental tasks were not significantly correlated with each other. There are several potential reasons for this finding. First, it is possible that our tasks were not sensitive enough for correlation analysis. Our behavioral tasks were initially designed for functional imaging, and a more fine-grained behavioral study—such as one relying on production errors rather than a forced choice about matching videos—could probe these relationships more clearly. Such an approach also could have facilitated better resolution in our VBM analysis. Next, it is also possible that our range of performance was not large enough to provide adequate correlations. Third, the task demands of the distance and angle tasks, which had an additional working memory component, could be different from those in the loop task. Although the loop task also required participants to store spatial information in working memory, the setups were different and could make direct comparison difficult. However, the distance and angle tasks had the same task structure, but still did not demonstrate a correlation. Finally, it is possible that these tasks are truly independent from each other, and that tracking a location taps a different path integration ability than tracking translation or rotation. This idea is similar to the dichotomy between homing vector models (constant tracking of the home location) and configural models (tracking the shape of the outbound path) of path integration (Fujita et al., 1990; Klatzky et al., 1999). Humans appear to flexibly use both strategies (Wiener et al., 2011), and it is possible that the loop task and the distance/angle tasks reflect the use of these two strategies. Thus, the lack of correlations between the experimental tasks leaves open several questions that could be explored in future studies.

We also found that the path integration abilities were not significantly related to other measures of spatial ability, namely the Santa Barbara Sense of Direction Scale (SBSOD), the Road Map Test, and the Perspective-Taking Test. The navigational abilities tests used in this study focused primarily on landmark-based navigation, not path integration, with the exception of a few of the questions in the SBSOD. This result is consistent with the idea that path integration and landmark-based navigation are separate systems. However, in both humans and animals, path integration is often assumed to be a major contributor to building successful allocentric (world-centered) spatial representations, which could incorporate landmark information (Gallistel, 1990; Maguire et al., 1998; Byrne et al., 2007; Chrastil, 2013). The lack of a correlation suggests that landmark-based navigation abilities as tested here are different from the path integration abilities tested in this study. This tension between landmark systems and path integration systems needs to be explored further.

Finally, the lack of any correlations between performance in the distance task and gray matter volume was surprising. Again, it is possible that our behavioral measures were not sensitive enough to detect correlations. We found several clusters that did not reach the threshold for cluster correction, suggesting that these effects were weaker or could have benefited from additional power. Another possibility is that successful participants relied on different strategies and brain regions to complete this task, in which case the variability between participants could have washed out any effects.

## Conclusions

We correlated gray matter volumes with performance in two path integration paradigms: a complex path integration task that involved tracking a home location, and a paradigm that examined distance (translation) and angle (rotation) estimation. Performance on the path integration tasks was largely independent, and performance in each task was associated with different gray matter volumes, suggesting that path integration is composed of multiple task-specific abilities. Most notably, we found that participants with larger gray matter volumes in retrosplenial cortex, hippocampus, and medial prefrontal cortex performed better on the loop task. These findings provide novel evidence that differences in gray matter volume in these regions contribute to individual abilities in path integration, and support computational and animal models of prefrontal-hippocampal-retrosplenial contributions to spatial navigation.

## Synthesis

Reviewing Editor: Kate Jeffery, University College London Decisions are customarily a result of the Reviewing Editor and the peer reviewers coming together and discussing their recommendations until a consensus is reached. When revisions are invited, a fact-based synthesis statement explaining their decision and outlining what is needed to prepare a revision will be listed below. The following reviewer(s) agreed to reveal their identity: Hugo Spiers, Jan Wiener

The authors present a study examining correlations between gray matter volumes and performance in a number of path integration tasks. They found that performance in complex path integration was largely independent from performance in more simple distance and rotation tracking and that the different tasks were associated with different grey matter volumes.

The study presents interesting results. However, there are some issues that should be addressed:

- The chosen tasks may not be ideal for addressing the overall research question. The tasks used by the authors have obviously been designed for a functional brain imaging study and may not be sensitive enough for the correlational approach. This may also explain the surprising lack of associations between the tasks. To investigate correlations between grey matter volumes and path integration abilities, tasks that provide more fine grained behavioural data would be more suited. This issue could be discussed.

- Chance level is 50% for all tasks. Given that performance in the loop task is less than 60%, it would be good to report statistical analysis comparing performance against chance level.

- Related to the last point, it would also be sensible to compare performance of individual participants against chance level. Given that overall performance in the loop tasks was only 59%, I would assume that several participants did not exceed chance level performance? If that was true, the authors should report how many participants could not solve the task and state whether or not their data was removed from the further analysis?

- The analyses of the behavioural data is very rudimentary only reporting average performance for the different tasks. It would be interesting to learn more about the effects of the experimental manipulations (speed, overshoot/undershoot, etc) on behaviour.

- The authors state that the lack of relationship between the navigation abilities measures and the three path integration task suggest that path integration could be independent of landmark-based navigation. This statement should be explained in more detail. Are the authors arguing that the navigation abilities measures target solely landmark-based navigation? Moreover, that path integration ans landmark based navigation are typically seen as different navigation mechanisms anyway.

- The lack of association between the different path integration tasks is puzzling and deserves a more detailed discussion than stating that tracking of self-motion could involve different aspects of path integration and that path integration is comprised of multiple task-specific abilities.' I assume the authors are not suggesting that path integration is independent of translation and rotation tracking? Could the lack of correlations be explained by the very limited range of “above chance level performance” in the loop task? Another possible explanation may relate to the different task demands of the paradigms, specifically, the additional memory, comparison and decision making components associated with the translation and rotation tasks.

- The lack of any correlations between accuracy in the distance task and grey matter volume should be addressed explicitly in the discussion.

The authors include their power calculation in the in the manuscript methods. I think they should refer to Hartley and Harlow 2012 as an example giving justification as to why they might expect a power of 0.8 and a sample of 26 being sufficient.

Related to this, it is essential the authors report the sample size in the abstract for study with VBM.

Fig 2 A it would be very useful to briefly list the brain areas shown in the image in the caption.

“Worsley, C., 2001.” In the reference list is not the full citation.

Reference to a study by Hok et al., 2005 PNAS on goal coding would be useful to include in this article when discussing the role of medial PFC.

Typo on line 488 should be corrected: “..there was no the relationship between..”

Please include information about the details of the task so that readers do not need to consult another paper in order to understand this one.

Was the video taken from the first person perspective?

What was the radius of the circle and the angular speed of movement around it?

What details were visible in the scenes?

Line 351 - Statistics need details (type of test, parameter, degrees of freedom etc) - here and elsewhere

Line 380 - what were te R values of the correlations shown in fig 2?

Line 566 “Comprised of” should be “composed of” or just “comprised”
